# Relationship between Illness Perception and Depressive Symptoms among Type 2 Diabetes Mellitus Patients in China: A Mediating Role of Coping Style

**DOI:** 10.1155/2020/3142495

**Published:** 2020-10-15

**Authors:** Jiarui Li, Xiaohui Qiu, Xiuxian Yang, Jiawei Zhou, Xiongzhao Zhu, Erying Zhao, Zhengxue Qiao, Yanjie Yang, Depin Cao

**Affiliations:** ^1^Department of Medical Psychology, Public Health Institute of Harbin Medical University, Heilongjiang Province, China; ^2^Medical Psychological Institute of the Second Xiangya Hospital of Central South University, Hunan Province, China; ^3^Department of Medical Education Management, Public Health Institute of Harbin Medical University, Heilongjiang Province, China

## Abstract

**Objective:**

The aims of the present study were to investigate the prevalence of depressive symptoms among type 2 diabetes mellitus (T2DM) patients in China and to explore how coping style influences the relationship between illness perception and depressive symptoms.

**Methods:**

Nine hundred and thirty-nine T2DM patients were recruited from a grade 3 Class A hospital in Harbin, China, and asked to complete a demographic questionnaire as well as the Self-rating Depression Scale (SDS), Brief Illness Perception Questionnaire-Revised (IPQ-R), and Medical Coping Modes Questionnaire (MCMQ). Hierarchical linear regression analysis and the bootstrap method were preformed to examine if coping style influenced the relationship between illness perception and depression.

**Results:**

The majority of patients (73.59%) exhibited depressive symptoms, including 37.27% with moderate and 6.71% with severe depressive symptoms. Depressive symptoms were more frequent in patients with complications (*P* < 0.05). A resignation coping style partially mediated the influence of illness perception on depressive symptoms.

**Conclusions:**

Interventions to improve coping style may reduce the prevalence or severity of depressive symptoms among T2DM patients, potentially enhancing treatment adherence and clinical outcome.

## 1. Introduction

Diabetes is a chronic and progressive metabolic disease characterized by high blood sugar [1]. The global prevalence of diabetes is rising annually, reaching 467 million in 2019 [2]. China alone had an estimated 116.4 million diabetes patients as of 2019, the largest such population in the world. The vast majority of diabetes mellitus cases (~90%) are classified as type 2 diabetes mellitus (T2DM). Diabetes can damage the kidneys, eyes, heart, and peripheral nerves, leading to progressive disability and premature death. In 2019, it is estimated that more than 4 million patients aged 20–79 years died from diabetes-related complications.

In addition to physical impairments and early mortality, T2DM can also negatively impact mental health. Multiple studies have found more frequent depressive symptoms among patients compared to matched controls, although prevalence varies widely across populations (4.5%–74%) [3]. Depressive symptoms hinder dietary and treatment adherence, thereby impairing glycemic control, exacerbating symptoms [4], and reducing the quality of life [5]. Therefore, it is critical to identify factors influencing depression in diabetes patients for more effective comprehensive treatment.

Illness perception refers to an individual's cognitive and emotional representations of illness [6]. It is influenced by perception of illness identity (i.e., the extent to which the illness defines personal identity), cause of illness, duration of illness, consequences of illness, curability of illness, and emotional representations. Inappropriate perception of illness can lead to psychological disorders [7], and a recent study found that maladaptive illness perception was directly associated with depression in diabetes patients [8]. However, few studies have examined illness perception among T2DM patients in China [9, 10], and even less attention has been paid to the potential mechanisms underlying the relationship between illness perception and depression.

Individual coping style is an important mediator of the relationship between stress and mental health [11]. Coping style refers to the cognitive or behavioral strategies developed to manage psychological stress. A positive (adaptive) coping style helps individuals solve and deal with stress and thus is beneficial to psychological health. By contrast, a negative coping style does not reduce stress and usually results in poorer psychological health [12]. Few studies have examined the potential link between coping style and depression among patients with T2DM. Both Burns et al. [13] and Yasui-Furukori et al. [14] reported that coping style was associated with depression in patients with T2DM. However, there is little research on how coping style influences the relationship between illness perception and depression among diabetes patients.

The aims of the present study were to investigate the prevalence of depression, assess the relationship between illness perception and depression, and explore the role of coping style in the relationship between illness perception and depression in patients with T2DM. We hypothesized that coping style mediated the relationship between illness perception and depression in T2DM.

## 2. Methods

### 2.1. Subjects and Procedure

With random number table, we selected a Grade-A Tertiary Hospital in Harbin, the capital of Heilongjiang Province located in northeast China. A cluster sampling method was employed to recruit a total of 962 patients diagnosed with T2DM from the Department of Endocrinology of a hospital between June 2018 and September 2019. 939 patients (97.61%) completed the questionnaires. Subjects included 556 males and 383 females [mean age, 41.08 years; standard deviation (SD), 12.27; range, 20–70]. The data and medical histories of the patients were obtained from questionnaires and medical records. Patients were excluded if they had other psychiatric diseases (aside from depression) or endocrine diseases. The study was approved by the Ethics Committee of Harbin Medical University, and all patients provided written informed consent.

### 2.2. Assessment of Depression

The Self-rated Depression Scale (SDS) was employed to assess depressive symptoms. The SDS is a 20-item questionnaire scored from 1 to 4 as follows: 1 (a little of the time), 2 (some of the time), 3 (good part of the time), and 4 (most of the time). The total score was calculated as the sum of the 20 items, with a score < 50 indicating no depression, 50–59 mild depression, 60–69 moderate depression, and >70 severe depression. The SDS is widely used in China, with Cronbach's coefficients of 0.79.

### 2.3. Assessment of Illness Perception

The nine-item Brief Illness Perception Questionnaire (BIPQ) was employed to assess illness perception [15]. The BIPQ contains eight items evaluating the cognitive and emotional representations of illness, including consequences, timeline, personal control, treatment control, identity, coherence, causes, and emotions, with Cronbach's coefficients of 0.86. Items are scored from 0 to 10. The last item is an open-ended question asking respondents to list three causal factors. A total score of illness perception was calculated as the sum of the 8 items. A higher score indicates a more threatening attitude of diabetes.

### 2.4. Assessment of Coping Style

The Medical Coping Modes Questionnaire (MCMQ) is a 19-item self-report measure of coping style to illness [16]. The Chinese version of the MCMQ used in this study is a 20-item questionnaire adapted to the unique characteristics of Chinese culture. It consists of items probing three coping style domains: confrontation (eight items), avoidance (seven items), and resignation (five items), with respective Cronbach's coefficients of 0.69, 0.66, and 0.76.

### 2.5. Statistical Analysis

The SPSS package (version 20.0 for Windows) was used for all data analyses. Demographic factors were compared between depressed and nondepressed patients by the chi-square (*χ*^2^) test. Correlation analysis was used to assess the relationships among illness perception, coping style, and depression. Mediation analysis was used to examine if coping style (mediator variable) influenced the relationship between illness perception (independent variable) and depression (dependent variable), with sex, age, education, and presence of complications treated as concomitant variables. All continuous variables were centralized to eliminate multicollinearity before conducting the mediation analysis. The mediation effect was then tested by SPSS bootstrap analysis using 5,000 samples. All tests were two-sided, and statistical significance was set at *P* < 0.05.

## 3. Results

### 3.1. Prevalence of Depression among T2DM Patients

According to the SDS (Table 1), the majority of the T2DM patients in this cohort exhibited symptoms of depression (691 of 939, 73.59%), with 248 (29.61%) showing mild, 350 (37.27%) moderate, and 63 (6.71%) severe depressive symptoms. Furthermore, the prevalence of depressive symptoms was higher in patients with complications than in patients without complications (*P* < 0.05).

### 3.2. Illness Perception

According to the total BIPQ score (43.85) (Table 2), patients expressed a relatively high degree of concern regarding their illness (i.e., considered the illness as threatening). The lowest mean score of illness perception is treatment control.

### 3.3. Associations among Illness Perception, Coping Style, and Depression

Table 3 presents the results of the correlation analyses among illness perception, coping style, and depression. Illness perception and resignation coping style were positively associated with depression severity (*P* < 0.01), and the conditions for conducting a mediating analysis were satisfied. Hierarchical regression analysis (Table 4) revealed that illness perception was positively associated with both resignation coping style and depression (*P* < 0.05) and resignation coping style was positively correlated with depression severity (*P* < 0.05). These results suggested that the relationship between illness perception and depression was partially mediated by a resignation coping style and bootstrap tests (Table 5) indicated that this mediation effect was significant (95% CI: 0.0457–0.1204). The direct effect of illness perception on depression severity was also significant (95% CI: 0.0016–0.0410), indicating that illness perception influenced depression among T2DM patients via direct and indirect (coping style-mediated) pathways.

## 4. Discussion

To the best of our knowledge, this was the first study to investigate the relationship between illness perception and depression among type 2 diabetes patients in China. The majority of these patients exhibited depressive symptoms, and almost half exhibited moderate to severe depression. The prevalence of depression varies widely (from 4.5% to 74%) across patient populations [3], including in Asia. For instance, a study from Japan found that 29.9% of T2DM patients exhibited depressive symptoms [14], compared to 44.1% in Nepal [8] and only 6.1% among another study population in China [17]. This marked variation in prevalence may be due to differences in measurement instruments or clinical factors such as disease duration and severity. In our cohort, the prevalence of depression was substantially higher than in most previous studies, including other studies from China. Based on this finding, we suggest that comprehensive T2DM treatment should place greater emphasis on mitigating comorbid depression as such patients exhibit poorer sleep quality [18], lower adherence to self-regulation behavior [19], and higher incidence of diabetes-related complications [4], resulting in higher healthcare costs [20].

The BIQR revealed that these T2DM patients viewed their condition as chronic with substantial adverse impacts on daily life and so were more concerned about their disease than reported in previous studies [7]. Again, this discrepancy may be explained by cultural factors, current disease severity, or confidence in treatment. Indeed, cultural and social contexts are major determinants of illness perception. In addition, the severity of complications may negatively influence illness perception and this population was composed of inpatients requiring comprehensive treatment. Alternatively, these patients demonstrated high levels of self-control and treatment control, consistent with previous studies [7, 10].

Illness perception was significantly associated with depression in our T2DM studies as documented previously [8]. The patients in this study viewed diabetes as chronic with serious adverse impacts on daily life; therefore, they had more depressive symptoms. Illness perception has also been linked to diabetes distress [21], which in turn is related to the severity of depression [22]. Burns and colleagues found a cyclical relationship between diabetes distress and depressive symptom in patients with T2DM [23], indicating that patients who perceive greater symptom severity also tend to have more severe depression symptoms.

Illness perception appears to influence depression via two pathways, a direct pathway and an indirect pathway mediated by coping style. A resignation coping style was positively associated with depression and so can be considered a risk factor for depression among T2DM patients. Patients with a high level of illness perception were more likely to use a negative coping style, which in turn resulted in increased depression severity. According to the appraisal theory of stress by Lazarus and Folkman [24], an individual's coping style is dependent on how they evaluate threats. These T2DM patients perceived diabetes as a chronic disease and experienced uncomfortable symptoms with significant negative impacts on daily life. Therefore, they tended to cope by resignation, a style in which stressful situations are accepted without any effort at change [25]. Patients with lower expectations of recovery usually adopt a resignation coping style [16], characterized by a feeling of hopelessness, leading to depression. According to the common sense model of self-regulation, individuals generate personal beliefs about threats such as chronic illnesses that then influence how they deal with illness [6]. Many studies have indicated that illness perception can influence the coherence of self-management behaviors [26]; therefore, patients with T2DM who adopt a resignation coping style usually fail to comply with dietary requirements and regular blood sugar monitoring, which can exacerbate their condition and result in even more severe depression. However, illness perception can be changed by targeted intervention, which in turn can improve glycemic control [27]. Therefore, interventions aimed at forming a more appropriate perception of diabetes and adaptive coping style may improve treatment compliance by reducing depression.

This study has several limitations. First, the cross-sectional design precludes causal analysis. Second, responses may be biased due to reliance on self-reported questionnaires. Third, the patients were from a single region (Heilongjiang Province), so these findings may not be applicable to other regions of China or other countries as social and cultural factors play an important role in forming illness perception. Larger-scale, multicenter, prospective studies are required to confirm and extend these findings.

## 5. Conclusion

In summary, the present study suggests a high prevalence of depressive symptoms among type 2 diabetes mellitus patients in China, potentially due to frequent adoption of a resignation coping style that exacerbates the influence of illness perception on depressive symptoms. Interventions that form more adaptive illness perceptions and coping styles may therefore reduce depression in T2DM, resulting in improved treatment compliance and clinical outcome.

## Figures and Tables

**Table 1:** Comparison of depressive symptoms between different patients with diabetes.

Group	Depression (*n* = 691)	Nondepression (*n* = 248)	*χ* ^2^	*P*
Sex			2.823	0.098
Male	398 (71.58)	158 (28.42)		
Female	293 (76.50)	90 (23.50)		
*Marriage*			0.283	0.576
Married	608 (73.88)	215 (26.12)		
Single	83 (71.55)	33 (28.45)		
*Education*			5.954	0.114
Primary school	81 (80.20)	20 (19.80)		
Junior school	209 (73.33)	76 (26.67)		
Senior school	198 (76.15)	62 (23.85)		
University and above	203 (69.28)	90 (30.72)		
*Income (yuan/month)*			3.827	0.281
<1000	99 (73.88)	35 (26.12)		
1000-1999	148 (72.20)	47 (27.80)		
2000-4999	297 (74.43)	102 (25.57)		
≥5000	136 (68.00)	64 (32.00)		
*Complications*			8.193	0.005
Yes	226 (79.86)	57 (20.14)		
No	465 (70.88)	191 (29.12)		
*Family history*			0.636	0.728
Yes	215 (74.91)	72 (25.09)		
No	412 (72.66)	155 (27.34)		

**Table 2:** Descriptive statistics for illness perception in patients with diabetes.

	Mean	SD
Consequences	6.79	2.74
Timeline	6.58	2.87
Personal control	3.50	2.60
Treatment control	2.98	2.33
Identity	6.70	2.46
Concern	7.32	2.44
Illness coherence	3.14	2.36
Emotional response	6.83	2.51
Illness perception	43.85	7.20

**Table 3:** Correlation between illness perception, coping style, and depressive symptoms.

Variables	Illness perception	Confrontation	Avoidance	Resignation	Depression
Illness perception	—				
Confrontation	0.038	—			
Avoidance	0.005	0.095^∗∗^	—		
Resignation	0.152^∗∗^	-0.106^∗∗^	0.278^∗∗^	—	
Depression	0.165^∗∗^	-0.158^∗∗^	0.164^∗∗^	0.478^∗∗^	—

^∗∗^ *P* < 0.01.

**Table 4:** Mediation analysis of resignation coping style on the relationship between the illness perception and depressive symptoms.

Variables	*B*	SE	*t*	*P*
Illness perception on depression	0.182	0.039	4.709	≤0.001
Illness perception on resignation	0.049	0.010	4.689	≤0.001
Resignation on depression (indirect effect)	1.738	0.108	16.126	≤0.001
Illness perception on depression (direct effect)	0.097	0.035	2.813	0.005

**Table 5:** Bootstrap results for the mediation analysis.

Variables	Estimate	SE	LL95%CL	UL95%CL
Illness perception on depression (direct effect)	0.1082	0.0343	0.0016	0.0410
Resignation on depression (indirect effect)	0.0824	0.0188	0.0457	0.1204

## Data Availability

The availability of the underlying data can be asked by email.
